# Stabilization of Clay Soils Using a Lime Derived from Seashell

**DOI:** 10.3390/ma18122723

**Published:** 2025-06-10

**Authors:** Luis Carlos Suárez López, Juan Carlos López Ramos, Yamid E. Nuñez de la Rosa, Giovani Jordi Bruschi, Jair de Jesús Arrieta Baldovino

**Affiliations:** 1 Civil Engineering Program, Universidad de Cartagena, Cartagena de Indias 130015, Colombia; lsuarezl@unicartagena.edu.co (L.C.S.L.); jlopezr1@unicartagena.edu.co (J.C.L.R.); 2 Faculty of Engineering and Basic Sciences, Fundación Universitaria Los Libertadores, Bogota 110231, Colombia; 3 Graduate Program in Environmental Science and Technology, Federal University of Fronteira Sul, Erechim 99700-970, Brazil; gio.bruschi@gmail.com

**Keywords:** soil stabilization, alkali-activated binders, mollusk shell waste, geomaterials, clay soils

## Abstract

The valorization of mollusk shell waste offers a promising alternative to conventional binders in soil stabilization, contributing to circular economy strategies and improved solid waste management. This study aimed to evaluate the mechanical and microstructural behavior of clayey soil stabilized with Waste Seashell Lime (WSL), a binder produced by calcining crushed snail and mussel shells at different temperatures (700–900 °C) and durations (2–4 h). A recommended calcination condition (800 °C for 2 h) was selected based on thermogravimetric analysis (TGA), scanning electron microscopy (SEM), and energy dispersive X-ray spectroscopy (EDX) results. WSL was incorporated at 3%, 7%, and 11% by dry soil weight and activated using NaOH at molarities ranging from 0.5 to 2.0 mol/L. A total of 122 specimens were prepared and tested for unconfined compressive strength (UCS) after 7 and 28 days. The highest UCS (4605 kPa) was recorded for the mix with 11% WSL and 1.0 mol/L NaOH at 28 days. At lower contents (3% and 7%), WSL-treated soils outperformed those stabilized with Type III Portland cement (Type III PC) under the same curing conditions. SEM-EDS analysis revealed the formation of cementitious phases, such as C–S–H and C–A–S–H, and factorial ANOVA confirmed the statistical significance of the WSL content, curing time, and alkali concentration. These results confirm the research hypothesis and demonstrate that alkali-activated WSL, derived from marine shell waste, can serve as a technically viable binder while supporting circular economy principles and waste reuse practices.

## 1. Introduction

Portland cement and lime are widely used in the construction sector due to their effectiveness in material improvement and infrastructure development. However, their production is associated with significant environmental impacts, particularly greenhouse gas emissions [1,2,3]. One ton of cement is estimated to release approximately one ton of CO_2_, while lime production emits around 0.86 tons of CO_2_ per ton produced [4,5]. Despite these figures, both materials are still considered among the most viable options in construction applications. In this context, the need to develop more sustainable cementitious materials has gained increasing importance [6]. At the same time, various industries generate large quantities of waste that have begun to be studied as potential inputs for soil improvement, aiming not only to valorize these by-products but also to contribute to the reduction of CO_2_ emissions in the production of new binders [7,8,9,10,11]. Among these residues, the shells of mollusks, such as oysters and mussels, are abundantly available worldwide. The leading producers in this industry include China, Japan, Korea, and France [12], with China accounting for 80% of global oyster cultivation and generating more than 300 tons of oyster shell waste annually [13]. This availability has prompted their study as an alternative source of lime and supplementary cementitious material.

Several studies have shown that the main component of oyster shells is calcium carbonate (CaCO_3_), which can be transformed into calcium oxide (CaO) through high-temperature calcination, making it a viable lime source [14,15]. Marine shell waste has been considered a potential alternative binder in geotechnical applications [16]. Recent studies have demonstrated that calcined mollusk shells can effectively stabilize clayey soils due to their high calcium oxide (CaO) content.

To serve as an effective soil stabilizer, seashells must undergo calcination—a thermal treatment process whereby raw shells are heated to high temperatures to decompose calcium carbonate (CaCO_3_) into calcium oxide (CaO) through the release of CO_2_. Previous studies have demonstrated that calcination at temperatures between 800 and 900 °C for durations of 2 to 4 h typically ensures a near-complete transformation of CaCO_3_ into reactive CaO [17,18]. Moghimi et al. [19] utilized seashell ash (SSA) to stabilize a high-plasticity clay and determined that the optimal calcination conditions were 900 °C for 1 h. Under these parameters, the resulting ash exhibited a calcium oxide (CaO) content of 94.74%, indicating a highly reactive lime suitable for geotechnical applications.

Similar results have been reported for cockle and clam shells, which, upon high-temperature calcination, yield ash predominantly composed of CaO that subsequently hydrates to form portlandite [18]. Conversely, incomplete decomposition may occur when the calcination temperature is insufficient (e.g., around 700 °C) or the exposure time is limited, resulting in residual carbonate and reduced ash reactivity [20]. In one comparative study, calcination temperatures of 700, 800, and 900 °C were applied to oysters, periwinkle (sea snails), and land snail shells, revealing a direct correlation between higher temperatures and an increased CaO content in the resulting ash. These findings indicate that thermal processing at approximately 800 °C or higher for several hours is generally necessary to obtain highly reactive lime suitable for geotechnical applications.

These sustainable alternatives have shown performance comparable to, or even exceeding, that of conventional lime. Almuaythir et al. [17] confirmed that processed clam shells, which were crushed using a jaw crusher and calcined at 800 °C for four (4) hours in a laboratory furnace, combined with silica fume and a small amount of lime, significantly enhance soil strength and compressibility, increasing the unconfined compressive strength (UCS) from approximately 115 kPa to 1510 kPa and reducing the plasticity index by up to ~75%. Likewise, Suwito et al. [21] demonstrated that incorporating cockle shell ash with silica fume decreased the liquid limit and improved compaction behavior. Anggraini et al. [22] achieved significant gains in strength and ductility using a blend of calcined shell ash and coir fiber in tropical lateritic soils. The optimal mix, comprising 9.06% CSS, 0.30% CF, and 12 days of curing, increased the unconfined compressive strength (UCS) from 153.11 kPa (untreated soil) to 1070.98 kPa, the flexural strength (FS) from 125.15 kPa to 572.09 kPa, and the indirect tensile strength (ITS) from 137.98 kPa to 585.32 kPa, representing improvements of approximately 7×, 4.5×, and 4.2×, respectively.

In line with these findings, Zhang et al. [23] proposed partially replacing cement with calcined oyster shells, reducing binder consumption by up to 46% without compromising the mechanical performance. Jeong et al. [24] found that shell-derived CaO neutralized acidic dredged sediments and increased the shear strength by promoting cementitious precipitation. Other studies demonstrated that calcined and raw shell powders improve the bearing capacity and reduce permeability in marine clays and sediments [17,25]. Ruiz et al. [26] showed that even unprocessed crushed shells can act as granular additives, improving particle gradation, providing mechanical stabilization, and contributing to chemical buffering in problematic sandy subgrades. These findings collectively support marine shell waste’s viability as an environmentally responsible alternative for soil improvement applications. The study by Gharzouni et al. [27] further supports this perspective by demonstrating that up to 40% of metakaolin can be replaced by shell waste without compromising the structural integrity.

Several studies have demonstrated that calcined seashells can serve as effective precursors in alkali-activated systems, offering a sustainable alternative for binder formulation. Hasnaoui et al. [28] achieved compressive strengths of up to 22 MPa with a porosity of 16.5%, while Wu et al. [16] reported a 30% improvement in the compressive strength and a comparable reduction in total porosity by partially replacing fly ash with seashell powder. Monneron-Gyuritsa et al. [12] showed that shells activated with metakaolin promote the formation of portlandite and C(A)SH phases, and Zhang et al. [23] confirmed their capacity to densify the concrete microstructure. These findings reinforce the potential of calcined seashells as alkali-activated binders. While natural shells act mainly as inert fillers, calcined shells actively participate in hydration reactions, forming phases such as portlandite and C(A)SH. The system’s effectiveness largely depends on the shell type and its calcium content, underscoring reactivity as a key variable in the performance of alkali-activated materials.

It has been established that mollusk shells can be used for lime production due to their high calcium carbonate (CaCO_3_) content, making them a potentially reactive raw material for soil stabilization applications. However, it remains necessary to determine under which specific conditions these calcined seashells can generate binder products capable of achieving strength levels comparable to those obtained with traditional binders such as Portland cement. In this context, the present research is based on the hypothesis that lime derived from seashells, when properly alkali-activated, can deliver mechanical and microstructural performance equivalent to or better than that of conventional cement, provided that the production and activation parameters are optimized.

This study evaluates the mechanical and microstructural behavior of waste seashell lime (WSL) in stabilizing clay soil from the northern area of Cartagena de Indias, Colombia. Cartagena is a coastal city where a wide variety of mollusk shells are abundantly available due to both local biodiversity and their frequent consumption by residents and tourists, making seashell waste a readily accessible and underutilized resource.

The WSL was alkali-activated using sodium hydroxide and compared to soil–cement mixtures prepared with Type III PC, under identical mixing and curing conditions. Locally sourced mussel and snail shells were crushed and calcined at varying temperatures (i.e., 700, 800, and 900 °C) and durations (2, 3, and 4 h) and selected based on their calcium content, which was determined through SEM-EDS. The resulting lime was then hydrated for use. WSL was applied at dosages of 3%, 7%, and 11% using dry soil weight activated with NaOH solutions at molarities ranging from 0.5 to 2.0 mol/L. The mixtures were compacted statically into cylindrical specimens and cured for 7 and 28 days. Specimens stabilized with Type III PC (i.e., 3%, 5%, 7%, 9%, and 11%) were also prepared for comparison. Unconfined compressive strength (UCS) tests were conducted, and SEM-EDS analyses were performed to characterize the microstructural features and identify the reaction products formed during stabilization.

## 2. Materials and Methods

The present study was structured into three phases. In the first phase, the physicochemical properties of the materials involved in the experimental design were characterized, i.e., clay, WSL, the alkaline activator (NaOH), and the cement used. The second phase focused on determining the new seashell-waste-based stabilizer’s optimal or recommended production conditions. Finally, a series of unconfined compressive strength tests were designed and conducted to analyze and compare the performance of this new binder against conventional Portland cement.

### 2.1. Materials

**Figure 1:**
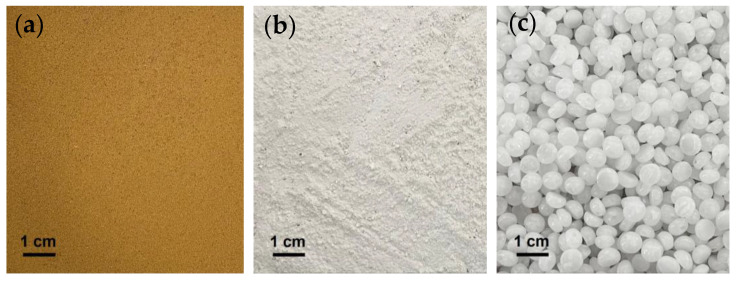
Materials studied: (**a**) clay; (**b**) WSL; (**c**) alkaline activator (NaOH).

This research utilized a clayey soil (Figure 1a) stabilized with a binder derived from lime (Figure 1b), produced through the crushing, calcination, and hydration of WSL (obtained from snail and mussel shells). The WSL was alkali-activated using an aqueous solution incorporating sodium hydroxide (Figure 1c) dissolved in distilled water. Type III PC was also included as a conventional stabilizing agent for comparative purposes.

#### 2.1.1. Soil Sample

**Figure 2:**
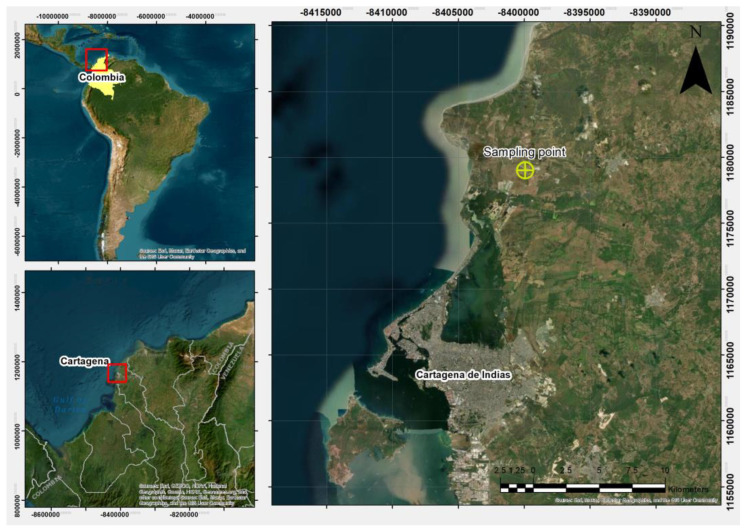
Geographic location of the sampling point for the studied clayey soil.

The soil studied originated from an urban expansion area north of Cartagena de Indias, Colombia, specifically at the geographic coordinates 10°30′21.8″ N, 75°28′27.1″ W (Figure 2). Additional characteristics of the site were described by Acuña et al. [29], who previously determined the dispersivity level of this clay using the pinhole test and the crumb test.

**Figure 3:**
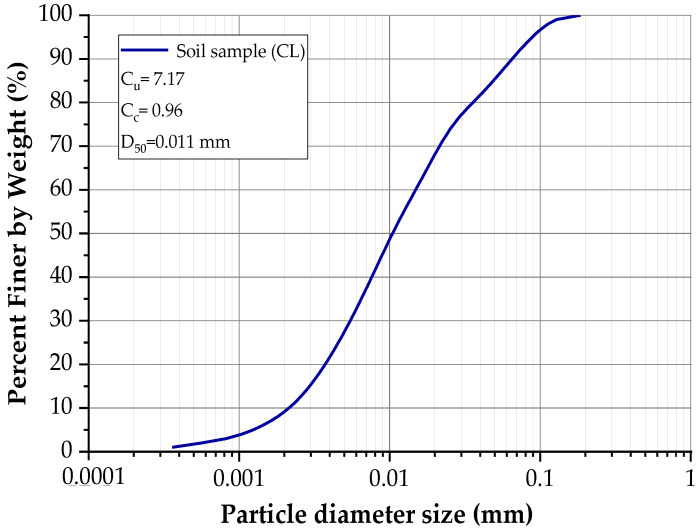
The granulometric curve of the soil sample.

**Figure 4:**
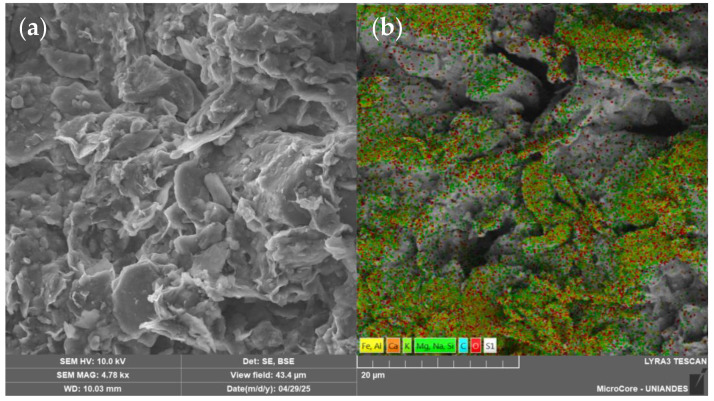
SEM images of the soil sample: (**a**) surface morphology captured in secondary electron (SE) mode; (**b**) backscattered electron (BSE) image with superimposed elemental mapping of the soil composition.

This clay was also stabilized using construction industry waste and a biopolymer According to these studies, the mineralogical analysis identified kaolinite as the predominant clay mineral [30,31]. Table 1 summarizes the physical properties of the soil, while Table 2 presents its chemical composition. Figure 3 shows the particle size distribution curve obtained through laser diffraction analysis. Additionally, Figure 4a illustrates the clay morphology observed via scanning electron microscopy (SEM) at a magnification of 4780 times, whereas Figure 4b displays the corresponding elemental mapping, highlighting the surface distribution of key elements such as silicon and aluminum.

#### 2.1.2. Waste Seashell-Derived Lime

Both snail shells and mussel shells consist predominantly of CaCO_3_ (≈95–98%), and upon calcination (typically around 700–900 °C for 1–6 h), they yield CaO (lime). Characterizations like XRD, SEM, TGA, and XRF confirm that calcined snail and mussel shell ashes are highly calcium-rich (often >90% CaO), explaining their similar reactivity and effectiveness as lime substitutes in soil stabilization, cement/mortar production, and other material applications. This evidence shows the compatibility of both snail and mussel shells for producing reactive limes in mixed formulations [17,36,37,38,39,40].

**Figure 5:**
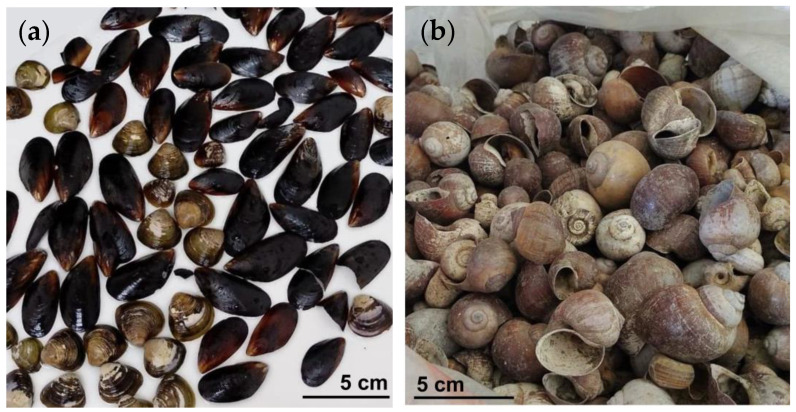
Seashells are used as a source of lime (WSL). (**a**) Mussel seashells; (**b**) snail seashells.

Produced waste from seashell-derived lime (WSL), mussel shells (Figure 5a), and snail shells (Figure 5b) were used as raw materials. These were collected by recycling seafood waste generated by restaurants in the historic center of Cartagena de Indias, Colombia.

To ensure the purity of the material and eliminate the residues of organic matter, dirt, and other impurities, the shells underwent a thorough washing process. Subsequently, they were oven-dried at a temperature of 100 °C for 24 h to remove residual moisture. Once dehydrated, the shells were crushed using the dynamic loading method, which allowed for the attainment of an appropriate particle size. Finally, the crushed material was sieved through a No. 40 mesh, a particle size considered suitable for its application in the next phase of the experimental study [41,42].

**Figure 6:**
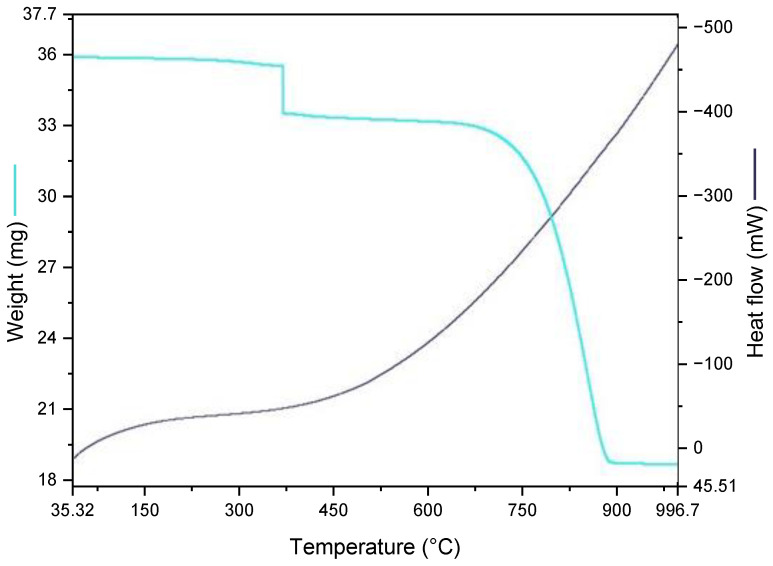
Thermogravimetric analysis (TGA) and heat flow curve of raw seashell powder. The light blue line represents mass loss (mg), and the dark line corresponds to heat flow (mW) as a function of temperature (°C).

Figure 6 presents the thermogravimetric (TGA) and heat flow curves obtained from raw seashell powder using a Pyris™ TGA analyzer (PerkinElmer series, Waltham, MA, USA).

The thermal program involved an initial hold at 35 °C for 1.0 min, followed by a continuous heating ramp from 35 °C to 995 °C at 20 °C/min under a controlled atmosphere. The TGA curve shows three distinct stages of mass change. An initial weight loss below 200 °C corresponds to the release of adsorbed moisture. A slight decrease around 400 °C may be attributed to the decomposition of organic residues or trace hydrated compounds. The main decomposition occurs between 700 °C and 900 °C, associated with the breakdown of calcium carbonate (CaCO_3_) into calcium oxide (CaO) and CO_2_, as supported by similar findings in the literature [16,28].

**Figure 7:**
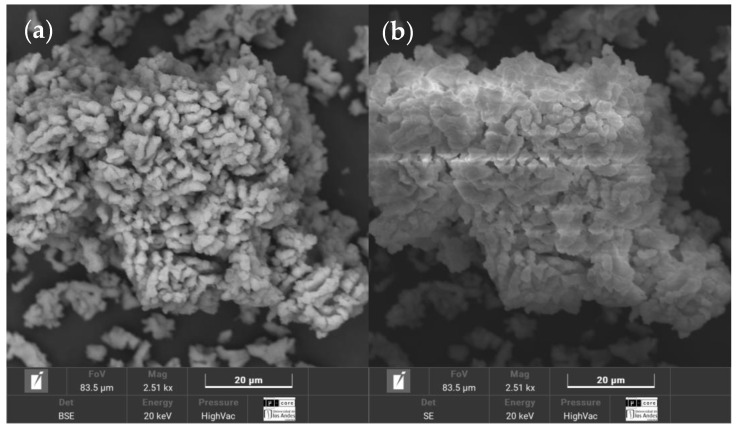
SEM morphology of lime WSL calcined at 800 °C for 2 h: (**a**) BSE image showing compositional contrast; (**b**) SE image highlighting surface topography.

To determine a recommended calcination temperature and duration for seashell-derived lime, both literature guidance and thermal analysis were considered [17,18,19,20]. Nine calcination profiles were evaluated, involving temperatures of 700 °C, 800 °C, and 900 °C, and durations of 2, 3, and 4 h. The thermogravimetric analysis (TGA) indicated that the main decomposition of calcium carbonate (CaCO_3_) occurs between 700 °C and 900 °C, with a pronounced endothermic peak near 800 °C, confirming the release of CO_2_ and formation of calcium oxide (CaO). This temperature was therefore identified as a critical threshold for effective calcination. To complement this, representative samples from each condition were analyzed via SEM-EDS. Figure 7 shows the morphology of the material calcined under this recommended condition.

**Figure A1:**
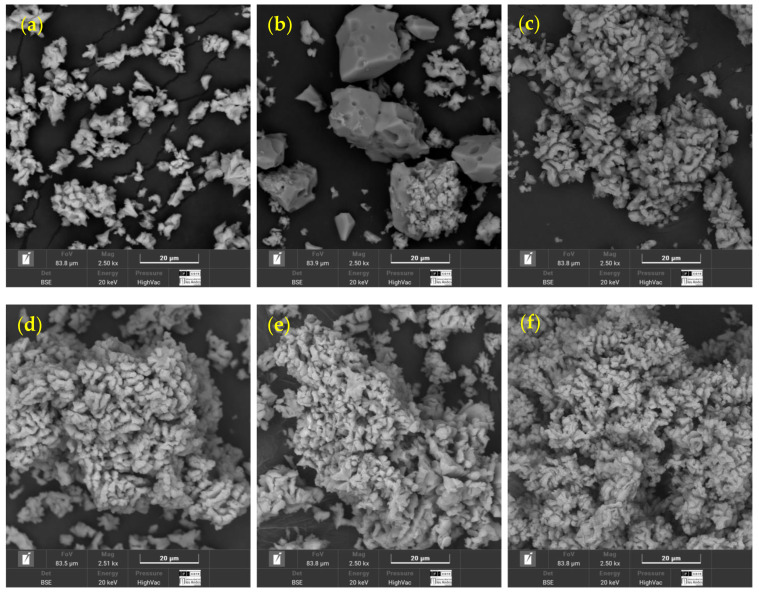

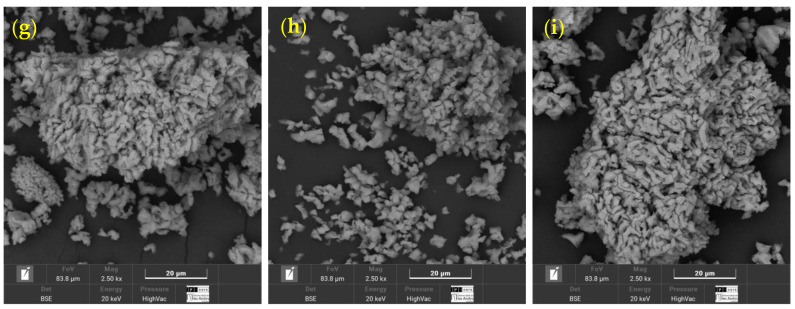
SEM micrographs of seashell waste after calcination at three temperatures (700 °C, 800 °C, and 900 °C) and three durations (2 h, 3 h, and 4 h). Each image was captured at 2.50 k× magnification, with a 20 µm scale bar and a field of view of 83.9 µm; (**a**) 700 °C–2 h, (**b**) 700 °C–3 h, (**c**) 700 °C–4 h, (**d**) 800 °C–2 h, (**e**) 800 °C–3 h, (**f**) 800 °C–4 h, (**g**) 900 °C–2 h, (**h**) 900 °C–3 h, (**i**) 900 °C–4 h.

Although SEM-EDS does not distinguish chemical phases, the calcium content and observed microstructural features served as indirect indicators of CaCO_3_ conversion and lime reactivity. Among the conditions tested, the combination of 800 °C for 2 h consistently exhibited favorable results. Detailed findings are presented in Appendix A, which includes SEM micrographs (Figure A1) and a summary of calcium concentrations for each condition (Table A1).

#### 2.1.3. Sodium Hydroxide

Sodium hydroxide (NaOH) pellets were used, with a reported purity of 99.6% (EMSURE^®^, ref. 1.06498.1000, batch MB2172698). According to the certificate of analysis (dated 17 March 2023), the material contains 0.2% carbonate, less than 0.012% chloride, and trace levels of heavy metals below 0.0005%, complying with high analytical-grade standards. The molar mass considered was 40 g/mol. The reagent was packaged in 1 kg containers and will remain stable until March 2026.

Four sodium hydroxide solutions with different concentrations (0.5 M, 1.0 M, 1.5 M, and 2.0 M) were prepared, each with a volume of 300 mL using distilled water. These solutions served as the alkaline activator in the mixture formulation of the test specimens. The activator was designed to stimulate the lime derived from mollusk shell residues chemically, enhancing its reactivity in the stabilization process.

### 2.2. Methodology

#### 2.2.1. Specimen Molding and Preparation

A total of 122 cylindrical specimens were fabricated according to the factorial design shown in Table 3. Of these, 50 were prepared with soil–cement mixtures, while the remaining 72 corresponded to soil stabilized with waste shell lime (WSL). For the WSL group, binder contents of 3%, 7%, and 9% by dry weight of soil were used, combined with four levels of activator molarity. The compaction dry density was kept constant at 17.6 kN/m^3^.

Specimens were molded with dimensions of 20 mm in diameter and 40 mm in height, respecting the 1:2 height-to-diameter ratio specified in ASTM D1631 [43] and commonly adopted in previous studies [44,45]. Static compaction was carried out using a hydraulic press under the strict control of mass and volume, applying rigorous rejection criteria to ensure dimensional and density consistency [46,47].

The water content was set at 18%, based on the soil’s optimum moisture content. The mixing procedures involved the manual homogenization of the dry components (soil and binder) and gradually adding the liquid phase. In WSL-treated mixtures, this consisted of an alkaline activating solution, whereas only distilled water was used for cement-treated specimens.

The specimens were cured in a humidity-controlled chamber at approximately 23 °C, and the relative humidity was close to 95%. To minimize moisture loss and ensure uniform curing conditions, each specimen was individually wrapped in a plastic sealing material as a moisture barrier.

To ensure specimen quality, strict acceptance criteria were enforced, restricting diameter and height variations to within 0.5 mm and 1 mm, respectively, from the target dimensions. Additionally, the mass of each individual specimen could not exceed 2% of the group’s average mass. Ultimately, strength results deviating more than 10% from the group average were excluded.

#### 2.2.2. UCS Test

Twenty-four hours before the completion of the curing period (on day 6 or day 27, depending on the case), the specimens were submerged in distilled water for approximately 24 h. This procedure aimed to reduce the effects of matric suction, as per previous studies’ recommendations [46,47,48].

The cylindrical specimens were tested for unconfined compressive strength (UCS) following the ASTM D2166 standard [49]. The tests were conducted using a 50 kN-capacity hydraulic testing press equipped with a sensitivity of 0.1 kN. Axial loading was applied at a constant deformation rate of 1.15 mm/min, ensuring controlled and consistent strain application throughout each test.

#### 2.2.3. Graphical and Statistical Analysis of the Influence of the WSL Content, NaOH Molarity, and Curing Time on the Strength of Stabilized Clay Soils

The contour plots, bar charts, and box-and-whisker diagrams were generated using OriginPro version 2025. This software facilitated the graphical representation of the UCS trends as a function of the WSL content, NaOH molarity, and curing time. All graphs were complemented with statistical indicators such as means and standard deviations to enhance the interpretation of mechanical behavior under different treatment conditions.

An analysis of variance (ANOVA) was conducted on the unconfined compressive strength results of the mixtures stabilized with WSL, using IBM SPSS Statistics version 29, with a significance level of 5% (*p* < 0.05). The analysis focused on evaluating the influence of the main factors—WSL content, NaOH molarity, curing time—and their interactions. This statistical evaluation made it possible to determine whether there were significant differences in the average strength values among the various experimental groups considered.

#### 2.2.4. Microstructural Analysis

After the UCS tests, selected compacted specimens of soil–cement and soil–WSL mixtures were prepared for a microstructural analysis (specimens with 7% and 11% cement, and in the case of WSL, 7% and 11% with varying sodium hydroxide molarities), using a Tescan LYRA-3 SEM-FIB system (TESCAN ORSAY HOLDING, Brno–Kohoutovice, Czech Republic). The samples were dried at 60 °C for 24 h, mounted on aluminum stubs with conductive carbon tape, and gold-coated when required. Imaging was performed at 15–20 kV using SE and BSE detectors, while EDS was applied to identify the elemental composition across targeted regions of each matrix.

## 3. Results

### 3.1. Effect of Waste Shell Lime Content, Alkaline Activator Molarity (NaOH), and Curing Time on the Unconfined Compressive Strength of Compacted Mixtures

**Figure 8:**
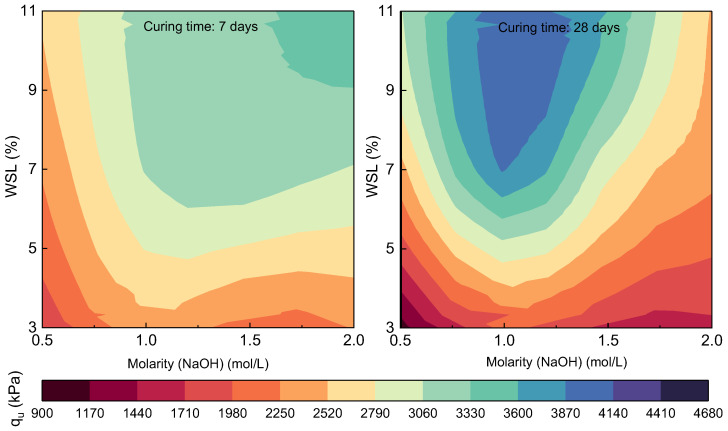
Contour maps of UCS of compacted mixtures as a function of the alkaline activator molarity (X-axis) and WSL % (Y-axis). The left panel shows results after 7 days of curing, and the right panel after 28 days, where the behavior with 1 mol/L NaOH stands out.

The mechanical behavior of the compacted mixtures treated with mollusk-shell-derived lime (WSL) was analyzed based on three key variables: the WSL content (3%, 7%, and 11%), the molarity of the alkaline activator (NaOH: 0.5 M, 1 M, 1.5 M, and 2 M), and the curing time (7 and 28 days). The primary parameter was the unconfined compressive strength (UCS), expressed in kPa. The results are presented in the contour plots of Figure 8, which illustrate how the UCS varies with the WSL content and molarity, comparing short-term (7 days) and medium-term (28 days) performance.

According to the data, the 1 M NaOH molarity yielded the most consistent and highest strength values at 28 days, particularly when combined with the 11% WSL content. Under this condition, mixtures reached UCS values of up to 4605 kPa, significantly higher than those observed with other molarities, even the higher ones. Compared to the mix with 3% WSL and 0.5 M (averaging around 1050 kPa), this represents an increase of approximately 338%, reflecting a strong synergistic effect between the biogenic binder content and the controlled alkaline activation.

At 7 days of curing, the 1 M molarity also showed strong performance. Mixtures with 7% and 11% WSL activated with 1 M reached UCS values between 3726 and 4241 kPa, clearly surpassing those obtained with 0.5 M and showing even greater consistency than mixes activated with 1.5 or 2 M, which showed more variable behavior. This early-age performance suggests that 1 M provides sufficiently effective activation from the first curing stages without adverse effects such as premature hydration or unstable phase crystallization.

The comparison between 7- and 28-day curing periods confirms the importance of time in strength development. In nearly all combinations, a progressive increase in UCS was observed as the curing time advanced. For instance, the mixture with 7% WSL and 1 M increased from 3757 kPa to 4242 kPa, representing an improvement of approximately 12.9%. This behavior indicates the ongoing pozzolanic reactions and the gradual structural consolidation of the solid matrix. Furthermore, in mixtures with lower WSL contents, the curing time effect was even more pronounced, reaffirming the critical role of proper curing in ensuring the full efficiency of the activation process.

### 3.2. Comparison of the Efficiency of Clay Soil Stabilization Using Alkali-Activated WSL and Type III PC Cured at 7 and 28 Days

**Figure 9:**
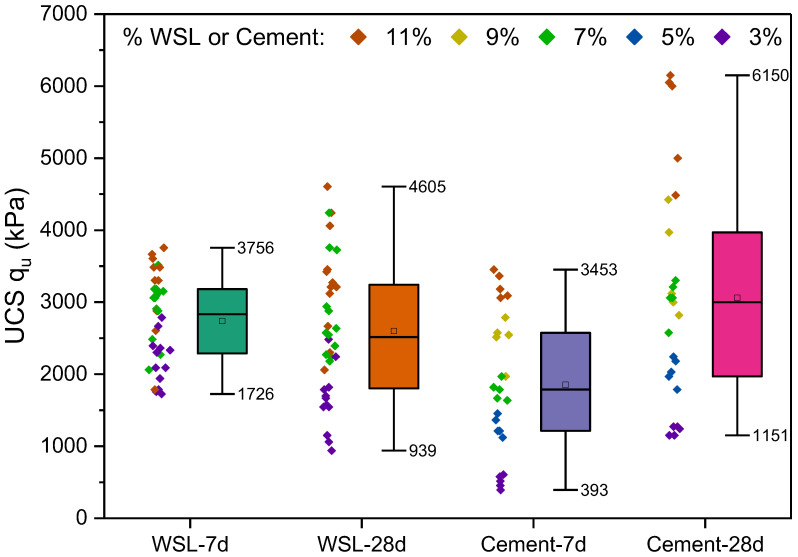
UCS (kPa) of soils stabilized with WSL (3%, 7%, 11%) and Type III PC (3%, 5%, 7%, 9%, 11%) at 7 and 28 days of curing. Each WSL group (i.e., 7-day and 28-day) includes 36 specimens, while PC-stabilized groups comprise 25 specimens per curing time.

Figure 9 compares unconfined compressive strength results across four groups of mixtures: soils stabilized with WSL cured at 7 and 28 days and soils stabilized with Type III PC under the same curing periods. According to the factorial experimental design, the WSL groups include binder contents of 11%, 7%, and 3% relative to the dry weight of the soil without distinguishing the molarity of the alkaline activator used. In the case of cement, the exact percentages were evaluated, along with additional dosages of 9% and 5%.

The unconfined compressive strength results reveal apparent differences between mixtures stabilized with WSL and those stabilized with Type III PC. For the 7 days of curing, WSL-stabilized specimens showed superior performance in terms of the minimum strength, reaching 1726 kPa compared to 393 kPa for cement, a more than 339% improvement in the minimum value. Even at a 3% dosage, WSL achieves strengths equal to or greater than those obtained with 7% cement, highlighting its early-age efficiency, especially at low binder contents.

However, at 28 days, Type III PC demonstrates a more pronounced and sustained strength gain. The maximum strength with 11% cement reaches 6150 kPa, while WSL peaks at 4605 kPa, giving PC a 33% advantage in peak strength. Additionally, the minimum strength with PC (1151 kPa) exceeds that of WSL (939 kPa) by 22%, indicating more consistent development. In the case of WSL, only the 11% mixtures show an increase in strength between 7 and 28 days; for lower dosages (3%), a relative decrease of up to 18% is observed. Therefore, alkali-activated WSL is advantageous for applications requiring early strength development, whereas PC is more effective for long-term soil stabilization processes with progressive mechanical performance.

**Figure 10:**
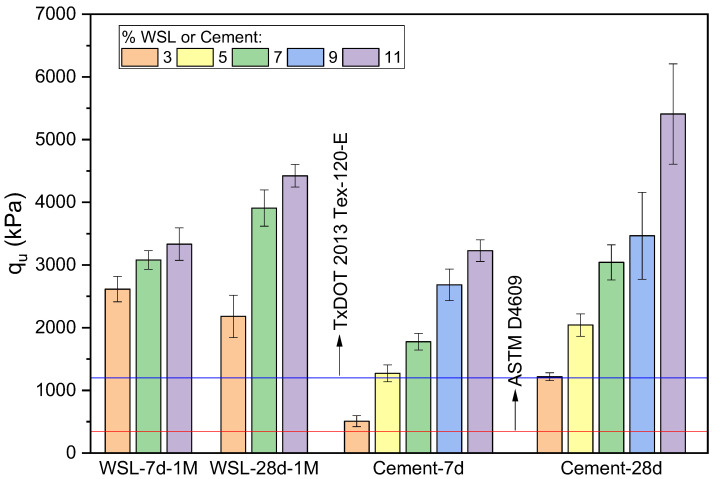
UCS (kPa) of soil stabilized with WSL-1M and Type III PC at 7 and 28 days of curing. Specimens with variation > 15% from the mean were discarded; final standard deviations are shown as error bars.

According to the experimental strength results, the alkaline activator with a molarity of 1 mol/L yielded the best performance compared to the other concentrations. Therefore, Figure 10 compares these results at 7 and 28 days of curing against the same values obtained from clay stabilized with Type III PC. The findings align with those shown in Figure 9, confirming that, at 7 days, the soil treated with WSL exhibits significantly better performance than the cement-stabilized soil, particularly at lower binder contents. However, this advantage decreases as the binder percentage increases. A similar trend is observed at 28 days: while WSL-treated samples outperform cement at 3% and 7% binder contents, the cement achieves higher strength at 11%, albeit with greater variability in the test results.

### 3.3. Statistical Analysis of the Influence of WSL Content, NaOH Molarity, and Curing Time on the Strength of Stabilized Clay Soils

The ANOVA performed for the unconfined compressive strength (q_u_) of the WSL-stabilized mixtures (Table 4) yielded a statistically significant model (*p* < 0.001), with an adjusted R^2^ of 0.524. Among the main factors, the WSL content exhibited the most decisive influence on strength development (*p* < 0.001), followed by the molarity of the alkaline activator (NaOH), which also had a significant effect (*p* = 0.002). In contrast, the curing time (7 and 28 days) did not show a statistically significant isolated impact (*p* = 0.285), although its interaction with molarity was significant (*p* = 0.048), suggesting a combined effect on the evolution of strength over time.

Despite the lack of isolated significance of the curing time, the experimental data demonstrate that strength development is sensitive to the interaction between the NaOH concentration and curing duration. For example, at an 11% WSL content, strength values increased from approximately 3363 kPa to over 4600 kPa as the molarity and curing time increased, indicating enhanced reactivity and the formation of cementitious products under optimized conditions. Similarly, at a 3% WSL, the strength gains between 7 and 28 days were limited or declined under suboptimal activation conditions, highlighting the system’s sensitivity to alkaline availability.

These findings underscore the critical role of the binder dosage and alkaline activation in optimizing the mechanical performance of WSL-stabilized soils. While the WSL content drives the overall strength, molarity modulates the activation kinetics, and their synergy appears to be essential, especially at early curing stages.

In Table 4, the superscript “a” associated with the corrected model sum of squares (28600608.958 ^a^) indicates that the model includes all main effects and interactions considered in the factorial design. The corrected R^2^ value was obtained using the adjusted R^2^ formula to account for the number of predictors in the model and avoid overestimating the explanatory power.

According to the classification proposed by Decky et al. [50], the coefficient of determination (R^2^ = 0.675) between the WSL content and unconfined compressive strength indicates a strong correlation, supporting the direct influence of this parameter on the mechanical performance of the stabilized soil. In contrast, the NaOH molarity (R^2^ = 0.064) and curing time (R^2^ = –0.089) show negligible correlations, suggesting that their effects are neither linear nor significant within the evaluated range. These findings align with the adjusted R^2^ value from the ANOVA model, reaffirming that the recycled lime content is the primary factor associated with strength enhancement.

### 3.4. SEM–EDS Analysis of the Stabilized Mixtures

**Figure 11:**
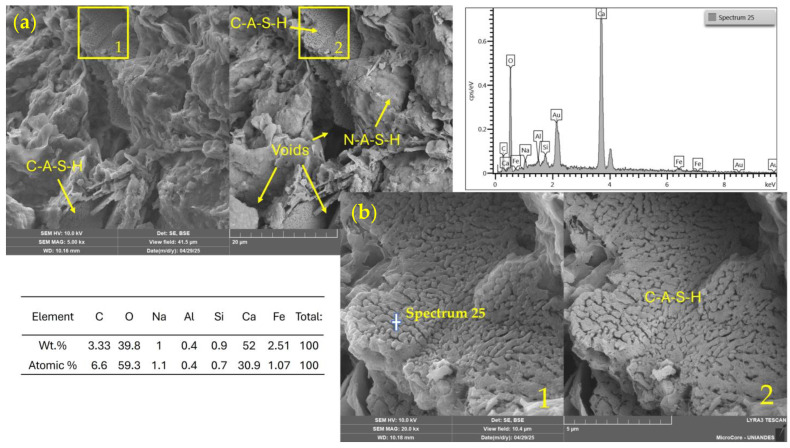
SEM/EDX images and elemental spectrum of a soil–WSL (7%)–NaOH 2 M stabilized mixture. (**a**) General view (field of view: 41.5 µm); (**b**) magnified area (10.4 µm). 1: SE detector; 2: BSE detector.

Figure 11a shows a heterogeneous microstructure of clay particles partially coated by geopolymeric or cementitious reaction products, such as C–S–H or C–A–S–H phases. Dense and bright agglomerates are visible, likely associated with calcium-rich compounds, including calcium silicate hydrate (C–S–H) or calcium carbonate (CaCO_3_), formed through the interaction between WSL and the soil’s native silicates. Microcracks and porous regions suggest an early stage of curing (7 days), where reaction products are still developing.

In Figure 11b, a magnified view of a dense area reveals a more compact matrix with visible crystal growth embedded in an amorphous phase. This morphology indicates the initial formation of cementitious structures triggered by the alkaline activation process. The observed development is consistent with reactions between calcium and the soil’s silica and alumina, promoting the gradual formation of stabilizing gels.

**Figure 12:**
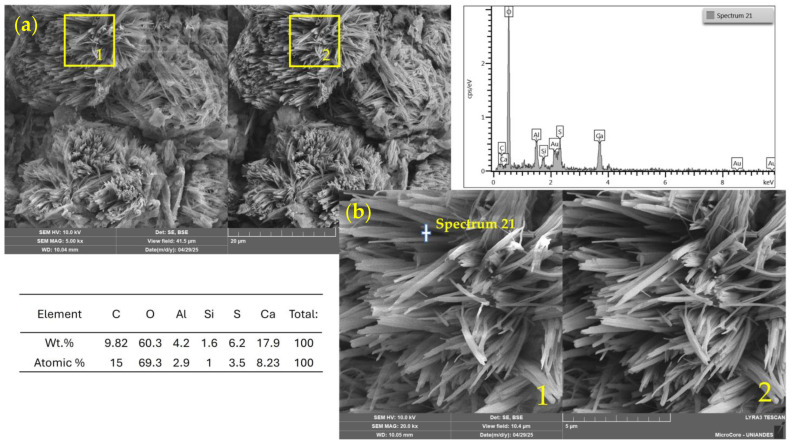
SEM/EDX images and elemental spectrum of a soil–WSL (7%)–NaOH 1.5 M stabilized mixture. (**a**) General view (field of view: 41.5 µm); (**b**) magnified area (10.4 µm). 1: SE detector; 2: BSE detector.

Figure 12, corresponding to a mixture with 7% WSL and 1.5 M alkaline activator, displays a heterogeneous microstructure with amorphous regions and dense agglomerates that suggest the formation of secondary reaction products. Notably, the EDS analysis revealed high concentrations of calcium (17.93 wt%) and sulfur (6.18 wt%), indicating the likely presence of calcium sulfate (CaSO_4_). This compound may result either from precipitation due to the high-alkaline environment after WSL incorporation or from the soil’s mineralogical composition, which contains 4% SO_3_, pointing to a natural gypsum or a sulfur-bearing origin.

Additionally, the detection of significant levels of carbon (9.82 wt%) and oxygen (60.29 wt%) supports the possible formation of calcium carbonate (CaCO_3_), typically associated with the carbonation of Ca(OH)_2_ in the presence of atmospheric CO_2_.

**Figure 13:**
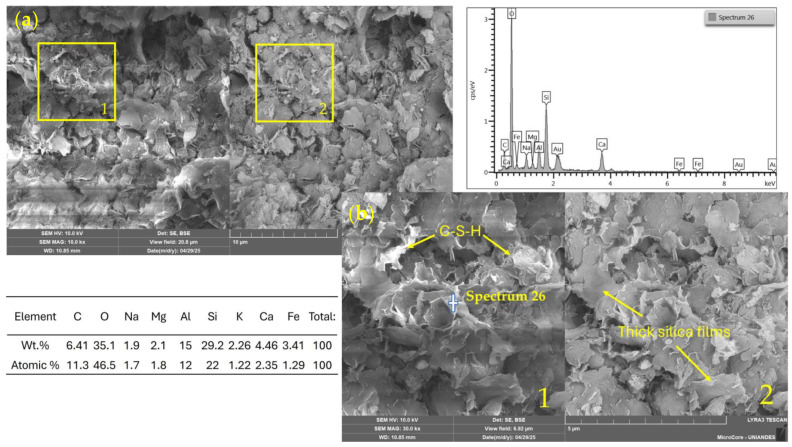
SEM/EDX images and elemental spectrum of a soil–WSL (11%)–NaOH 2 M stabilized mixture. (**a**) General view (field of view: 41.5 µm); (**b**) magnified area (10.4 µm). 1: SE detector; 2: BSE detector.

Figure 13 shows the SEM/EDS analysis of a mixture containing 11% binder activated with 2 M NaOH. The microstructure exhibits a dense and continuous matrix with low porosity and a notable presence of C–S–H phases, supported by a silica content of 29%, according to the EDS results. Thick silica films are also visible, possibly related to partially reacted gel products. The chemical composition suggests favorable mineralogical evolution, forming C–A–S–H-type phases induced by alkali activation.

**Figure 14:**
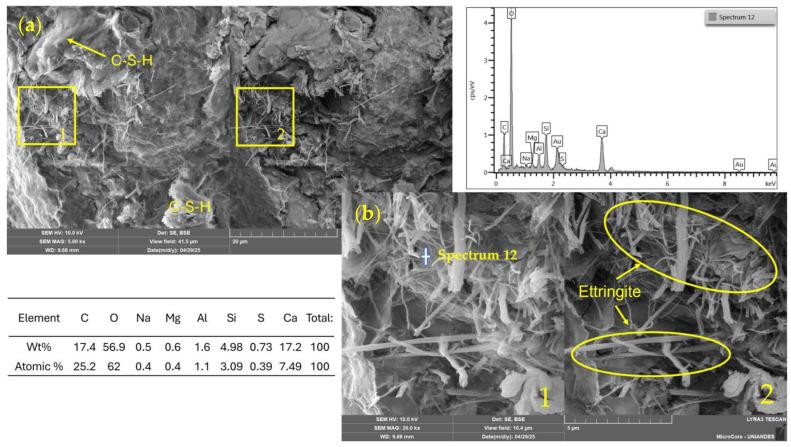
SEM/EDX images and elemental spectrum of a soil–cement (11%) stabilized mixture. (**a**) General view (field of view: 41.5 µm); (**b**) magnified area (10.4 µm). 1: SE detector; 2: BSE detector.

Figure 14, corresponding to a mixture stabilized with 11% CP, reveals a compact matrix with areas rich in hydration products. Gel-like continuous masses associated with C–S–H phases are distinguished, contributing to matrix cohesion and strength development. In addition, needle-like structures typical of ettringite are identified, formed through the reaction between tricalcium aluminate (C_3_A) from CP and the added gypsum under early hydration conditions.

The EDS analysis supports this interpretation, indicating high levels of calcium (17.2 wt%), silicon (4.98 wt%), aluminum (1.6 wt%), and sulfur (0.73 wt%)—elements characteristic of CP hydration products. The coexistence of these elements confirms the formation of primary ettringite, while the significant proportions of calcium and silicon reflect the dominant presence of C–S–H, the key phase responsible for the strength development in the soil–CP system.

## 4. Discussions

The results obtained in this study demonstrate a significant improvement in the unconfined compressive strength (UCS) of the clay stabilized with alkali-activated seashell lime (WSL). This enhancement is particularly notable when compared to the performance of the same soil treated with Type III Portland cement (PC), which served as the reference group in this investigation. While cement achieved UCS values above 3000 kPa only under high dosages (11%) and after 28 days of curing, the mixtures containing alkali-activated WSL reached comparable or superior strengths under equivalent conditions, particularly when 1.0 M NaOH was used. This highlights the effectiveness of the alkaline activation process on this CaO-rich by-product.

When comparing these findings with previous studies conducted by Baldovino et al. [31] on the same clay, where a biopolymer (Xanthan gum) reinforced with polypropylene fibers (PPF) was employed, the UCS values ranged between 600 and 1400 kPa depending on the mixture proportions. The strength values reported in that case were lower than those obtained with the activated lime mixtures in the present study, suggesting that the cementitious gels formed (such as C–S–H and C–A–S–H) offer a much more effective consolidation mechanism.

In another line of research, mixtures incorporating crushed glass (GG), gypsum (GY), or limestone waste (CLW) also yielded relevant mechanical gains. Nevertheless, even when these residues were blended with cement as the main binder, the UCS values ranged from 700 to 1900 kPa, depending on the porosity/binder index (η/B_iv_) [26,51].

From an environmental perspective, seashells represent an underutilized resource with high recovery potential. Approximately 10 to 20 million metric tons of shell waste are generated annually, accounting for approximately 22% of global aquaculture production [52,53,54]. Mussels contribute the highest volume of shells among bivalves, positioning them as a significant source of calcium carbonate (CaCO_3_) [55]. However, most of these shells are discarded as household or industrial waste without an integrated recovery strategy [56].

Far from being mere waste, these by-products can serve as valuable raw materials for soil stabilization. Recent studies emphasize that large-scale utilization depends on the availability of a stable supply and appropriate collection and processing systems [56]. While the thermal decomposition of shells generally requires temperatures above 1000 °C, as reported by Ishangulyyev et al. [57] and Kobatake & Kirihara [58], more energy-efficient alternatives such as enzymatic cleaning have been proposed. These methods reduce energy consumption and yield additional outputs such as protein hydrolysates. In this study, seashell calcination was carried out at 800 °C for 2 h—within the range recognized as optimal to activate their cementitious potential while maintaining process sustainability [59,60]. It has also been reported that the thermal treatment temperature influences the crystalline and amorphous content, affecting their reactivity [61].

Moreover, the use of calcined shells has proven effective in modifying soil pH and inducing the formation of calcium silicate hydrate (C–S–H) and calcium aluminate hydrate (C–A–S–H), which play a crucial role in immobilizing heavy metals in contaminated soils. This expands their potential use in environmental geotechnics [62]. At the microstructural level, fine calcined shell particles contribute to a denser matrix by filling voids and enhancing the internal structure of the treated soil [19].

Recent studies highlight that the use of these marine by-products contributes to the circular economy by valorizing abundant waste from the fishing industry and converting it into a useful construction material, thereby reducing the need for non-renewable raw materials [63,64]. Moreover, the valorization of this marine waste represents a sustainable management strategy, as it prevents tons of shells from ending up in landfills and reduces the environmental impact associated with the production of traditional binders such as cement [17,36].

In summary, the stabilization approach assessed in this study proves effective in terms of mechanical performance and aligns with circular economic principles. The alkali-activated seashell lime (WSL) emerges as a viable alternative to cement, particularly in the short term and at low dosages, while also incorporating a widely available waste material with demonstrable cementitious properties. These findings reinforce the role of WSL as a geomaterial solution for infrastructure development, foundations, and soil remediation applications.

## 5. Conclusions

This study confirmed the hypothesis that lime derived from mollusk shells, when properly calcined and alkali-activated, can achieve mechanical and microstructural performance comparable to, or even surpassing, that of conventional Portland cement, if production and activation parameters are adequately controlled.

- Based on thermogravimetric analysis (TGA-DTG) and SEM-EDS observations, a recommended calcination condition for producing reactive waste seashell lime (WSL) is 800 °C for 2 h. This temperature–time combination was shown to enable the effective decomposition of calcium carbonate into calcium oxide while avoiding issues such as over-calcination or sintering, which may negatively impact reactivity. These conditions are therefore proposed as a practical guideline for future studies aiming to value seashell waste in sustainable construction materials.
- In terms of mechanical behavior, the highest unconfined compressive strength (UCS) was observed with an alkaline activation of 1 mol/L NaOH and 11% WSL content, reaching values up to 4605 kPa at 28 days of curing. This performance outperformed Portland cement at lower binder contents (3% and 7%), demonstrating that WSL can be a technically viable and sustainable alternative, particularly for applications with material or cost constraints. However, at the 11% dosage level, Portland cement exhibited slightly higher UCS values, albeit with more dispersion.
- Microstructural analysis revealed dense matrices rich in cementitious gels such as C–S–H and C–A–S–H in WSL-stabilized samples, confirming the effectiveness of alkali activation as a mechanism for producing binding phases. These findings were further supported by factorial ANOVA, which demonstrated that the WSL content, curing time, and NaOH molarity significantly influenced the compressive strength (*p* < 0.05), including notable interaction effects. The combination of 11% WSL with 1 mol/L NaOH at 28 days provided the most consistent and robust performance, aligning with the overall experimental trends.

Despite these promising outcomes, the study has some limitations. All experiments were conducted under controlled laboratory conditions, and field-scale validation is necessary to confirm real-world applicability. Long-term durability aspects such as resistance to wet–dry cycling, chemical attack, and leaching behavior were not evaluated and remain areas for further research. Additionally, the scope was limited to a single clayey soil type, which restricts the generalizability of the findings.

As a future direction, combining WSL with other industrial by-products—such as fly ash or alkaline slags—may further improve performance while optimizing economic and environmental outcomes. Moreover, life cycle assessment (LCA) and techno-economic analyses are recommended to evaluate the feasibility and sustainability of this alternative. It is also advisable to explore the scalability of WSL production under industrial calcination systems and its integration into low-carbon construction practices, particularly in coastal regions with abundant mollusk shell waste.

## Figures and Tables

**Table 1:** Geotechnical properties of the soil sample.

Property of Soil	Standard	Value
Consistency limits		
Plasticity limit, P.L.	[32]	26.05
Plastic index, P.I.	[32]	15.95
Grain-size distribution		
Fine Sand (0.075–0.425 mm) %	[33]	12
Silt (0.002–0.075 mm) %	[33]	78
Clay (<0.002 mm) %	[33]	10
Mean diameter (*d*_50_) mm	[33]	0.011
Effective diameter (*d*_10_) mm	[33]	0.0021
Compaction characteristics (Standard Effort)		
Optimum moisture content (%)	[34]	18.2
Maximum dry unit weight (kN/ m^3^)	[34]	17.6
USCS Classification	[33]	CL
Specific gravity	[35]	2.8

**Table 2:** Chemical composition of soil sample (% by weight).

Materials	SiO_2_	Al_2_O_3_	SO_3_	K_2_O	CaO	Fe_2_O_3_	TiO_2_	LOI *
Soil (CL)	66	21.1	4	3.1	3	0.9	0.3	1.6

* Loss of mass through ignition.

**Table 3:** Mixed proportion design for compacted blends of soil, WSL, and cement.

Mix	Weight (%)			Molarity (NaOH) (mol/L)	Curing Time (d)	Number of Specimens
	Soil	Cement	WSL			
Soil–Cement	100	3	-	-	7, 28	10
	100	5	-	-	7, 28	10
	100	7	-	-	7, 28	10
	100	9	-	-	7, 28	10
	100	11	-	-	7, 28	10
Soil–WSL	100	-	3	0.5, 1, 1.5, 2	7, 28	24
	100	-	7	0.5, 1, 1.5, 2	7, 28	24
	100	-	11	0.5, 1, 1.5, 2	7, 28	24

**Table 4:** ANOVA table for the unconfined compressive strength (q_u_) results.

Source	Sum of Squares	Degrees of Freedom	Mean Squares	Z	*p*-Value	Significance (*p*-Value < 0.05)
Corrected Model	28,600,608.958 ^a^	17	1,682,388.762	5.592	<0.001	yes
Intersection	512,442,762.3	1	512,442,762.3	1703.215	<0.001	yes
WSL Content	18,393,803.36	2	9,196,901.681	30.568	<0.001	yes
Molarity (NaOH)	5,019,283.153	3	1,673,094.384	5.561	0.002	yes
Curing Time (t)	350,982.347	1	350,982.347	1.167	0.285	no
WSL * NaOH	1,489,206.972	6	248,201.162	0.825	0.556	no
WSL * t	809,789.528	2	404,894.763	1.346	0.269	no
NaOH * t	2,537,543.597	3	845,847.865	2.811	0.048	yes
Error	16,246,869.69	54	300,867.957			
Total	557,290,241	72				
Corrected Total	44,847,478.65	71				

* R^2^ = 0.638 (R^2^ corrected = 0.524).

## Data Availability

The original contributions presented in this study are included in the article. Further inquiries can be directed to the corresponding authors.

## Abbreviations

The following abbreviations are used in this manuscript:

WSL	Waste Seashell Lime
PC	Portland Cement
NaOH	Sodium Hydroxide
TGA	Thermogravimetric analysis
UCS	Unconfined Compressive Strength
q_u_	Compressive Strength
SEM	Scanning Electron Microscopy
EDS	Energy Dispersive X-ray Spectroscopy
C–S–H	Calcium Silicate Hydrate
C–A–S–H	Calcium Aluminum Silicate Hydrate
R^2^	Coefficient of Determination
SE	Secondary Electrons (SEM Detector)
BSE	Backscattered Electrons (SEM Detector)
wt.%	Weight Percent

## Appendix A

Appendix A provides complementary information to Section 2.1.1. Figure A1 shows SEM micrographs of the partially calcined seashell waste, corresponding to the nine combinations derived from the three calcination temperatures and three durations evaluated. All images were acquired using a BSE detector at a magnification of 2500×, with a representative scale bar of 20 µm and a field of view of 83.9 µm.

Table A1 summarizes the calcium content (wt.%) obtained from EDS measurements taken at different regions of the SEM images. The table reports the minimum, maximum, and average values, as well as the standard deviation for each calcination condition.

**Table A1:** Calcination temperatures and durations evaluated in the preparation of WSL.

Calcination Temperature (°C)	Duration (h)	Calcium Content (wt.%)			Standard Deviation
		Min	Mean	Max	
700	2	32.71	43.55	84.21	22.74
	3	27.10	36.52	48.72	11.08
	4	32.87	39.33	45.78	9.13
800	2	29.24	53.36	71.58	21.78
	3	31.06	50.64	63.21	17.18
	4	23.71	32.10	23.71	8.25
900	2	31.06	36.16	43.25	6.33
	3	28.77	37.11	50.07	11.37
	4	32.80	58.24	83.01	25.11
